# Inhibition of *in vitro* Ebola infection by anti-parasitic quinoline derivatives

**DOI:** 10.12688/f1000research.22352.1

**Published:** 2020-04-17

**Authors:** Shawn Goyal, Beth Binnington, Stephen D.S. McCarthy, Didier Desmaële, Laurent Férrié, Bruno Figadère, Philippe M. Loiseau, Donald R. Branch

**Affiliations:** 1 Department of Laboratory Medicine and Pathobiology, University of Toronto, Toronto, Ontario, M5H 2N2, Canada; 2 Centre for Innovation, Canadian Blood Services, Toronto, Ontario, M5H 2N2, Canada; 3 Institut Galien, CNRS, Université Paris-Saclay, 5 Rue Jean-Baptiste Clément, Chatenay-Malabry, 92290, France; 4 CNRS BioCIS, Université Paris-Saclay, Châtenay-Malabry, 92290, France; 5 Department of Medicine, University of Toronto, Toronto, Ontario, Canada; 6 Division of Advanced Diagnostics, Toronto General Hospital Research Institute, Toronto, Ontario, Canada

**Keywords:** Ebola virus, antiparasitic drugs, antiviral activity

## Abstract

There continues to be no approved drugs for the treatment of Ebola virus disease (EVD). Despite a number of candidate drugs showing limited efficacy *in vitro* and/or in non-human primate studies, EVD continues to plaque certain areas of Africa without any efficacious treatments yet available. Recently, we have been exploring the potential for anti-malarial drugs to inhibit an *in vitro* model of Ebola Zaire replication using a transcription-competent virus-like particle (trVLP) assay. We examined the efficacy of chloroquine, amodiaquine and 36 novel anti-parasite quinoline derivatives at inhibiting Ebola virus replication. Drug efficacy was tested by trVLP assay and toxicity by MTT assay. Both chloroquine and amodiaquine were effective for inhibition of Ebola virus replication without significant toxicity. The half-maximal inhibitory concentration (IC _50_) of chloroquine and amodiaquine to inhibit Ebola virus replication were IC _50, Chl _= 3.95 µM and IC _50, Amo _= 1.45 µM, respectively. Additionally, three novel quinoline derivatives were identified as having inhibitory activity and low toxicity for Ebola trVLP replication, with 2NH2Q being the most promising derivative, with an IC _50_ of 4.66 µM. Quinoline compounds offer many advantages for disease treatment in tropical climates as they are cheap to produce, easy to synthesize and chemically stable. In this report, we have demonstrated the potential of anti-parasite quinolines for further investigation for use in EVD.

## Introduction

Since its discovery in 1976, Ebola virus has been responsible for numerous outbreaks, with case fatalities varying from 20% to 90% ^1, 2^. From 2014 to 2016, West Africa was devastated with the largest Ebola outbreak in recorded history with six countries affected: Nigeria, Senegal, Guinea, Liberia, Mali and Sierra Leone. With no effective therapies or licensed vaccines available, little could be done to treat patients and prevent the spread of the virus ^3^. Over a period of two years, Ebola virus was responsible for 28,646 reported infections, with 11,323 deaths ^1, 3^.

Since the outbreak, a vaccine for Ebola virus (rVSV-EBOV) has been developed by the National Microbiological Laboratory in Winnipeg, Manitoba, Canada, and has been shown to be highly protective ^4^. Undoubtedly, the rVSV-EBOV vaccine is to be a powerful defense against future Ebola outbreaks. However, it is unlikely to be sufficient to stop future outbreaks. As the natural host of Ebola virus remains unknown, eradication of Ebola reservoir populations is impossible ^5^. Because of this, future spillover events are an inevitability ^6^. Additionally, the unstable political environment of many affected African countries further complicates the effective administration of vaccines; as seen in the Democratic Republic of Congo during the current Ebola outbreak ^2, 7^. These factors emphasize the need for acute infection therapies.

Previous publications have focused on potential drugs predicted to have efficacy against Ebola virus disease (EVD) and included drugs already approved and widely used that could perhaps be repurposed as treatments for EVD ^8, 9^. Through a virtual screening process, Veljkovic *et al.* ^8^ identified 267 US Food and Drug Administration (FDA)-approved drugs with possible inhibitory effects against Ebola virus. Among these were the antimalarial quinoline drugs amodiaquine and chloroquine. Chloroquine has been shown to be protective against Ebola virus disease in an *in vivo* mouse model ^9^. Additionally, amodiaquine has been identified as having inhibitory effects in a pseudo-type entry assay of Ebola virus ^9^. Furthermore, retrospective analysis of relative risks for patients from the 2014 to 2016 West Africa outbreak identified amodiaquine in combination with artesunate as having therapeutic effects. Data have suggested that patients infected with Ebola virus that were prescribed artesunate-amodiaquine had a significantly reduced risk of death compared to those that were prescribed artemether-lumefantrine, the latter treatment not being statistically significantly different from no treatment ^10^. This suggests that the quinoline derivative drug class may have therapeutic use in the treatment of EVD.

In this article, the efficacy of 36 novel quinolines ( Table 1) derivatives and previously approved quinoline compounds (amodiaquine and chloroquine) were examined for their ability to inhibit Ebola virus replication. Our results support previous reports that suggested that amodiaquine and chloroquine could be potential treatments for EVD. In addition, we identified additional, novel, quinolines that could be candidates for further study of their potential for inhibition of Ebola virus infection.

**Table 1.** Antiparasitic quinolines examined, chemical structures, inhibitory activity and toxicity testing.

Drug	Structure	Inhibition at 10µM	Viability at 10µM
Amodiaquine	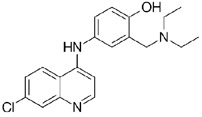	97.67± 10.62%	87.71± 1.00%
Chloroquine	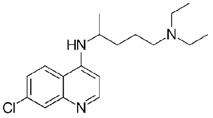	92.16± 6.23%	106.26± 1.24%
2NH2Q	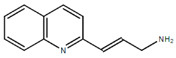	89.74± 6.53%	81.10± 2.09%
2PentQ	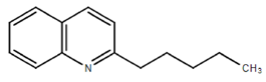	-6.39± 9.15%	103.48± 0.99%
2OHQ	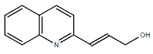	89.69± 4.09%	31.15± 1.27%
2COOHQ	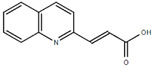	30.58± 6.15%	88.54± 1.54%
2CNQ	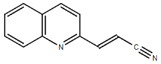	3.07± 8.34%	87.95± 3.63
2PQSel	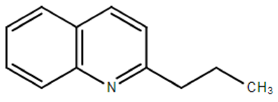	10.85± 32.36%	97.61± 2.06%
2QPOH	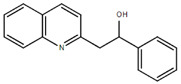	-113.56± 92.5%	88.33± 3.26%
2Q16OH	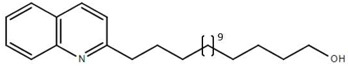	-123.88± 30.63%	74.05± 0.75%
2Qi15	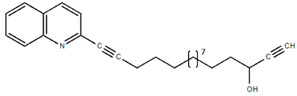	93.27± 4.94%	109.52± 0.25%
2QQ	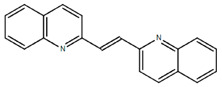	74.33± 13.45%	57.31± 0.29%
XF906	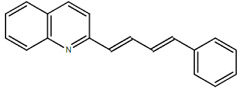	0.21± 20.27%	104.49± 1.65%
BS460	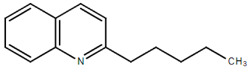	4.04± 44.15%	107.53± 0.59%
DD1	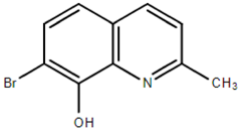	90.49± 8.32%	59.69± 0.03%
DD2	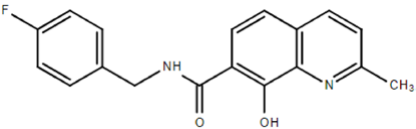	80.77± 1.38%	77.05± 4.78%
DD3	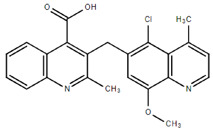	-156.04± 97.28%	105.49± 2.54%
DD4	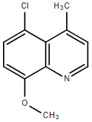	49.7± 1.17%	90.09± 1.64%
DD5	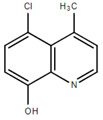	-66.45± 17.78%	51.44± 1.31%
MBN111	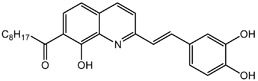	-48.86± 87.76%	42.50± 4.97%
MBN132	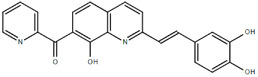	-10.40± 57.69%	104.57± 1.39%
MBN91	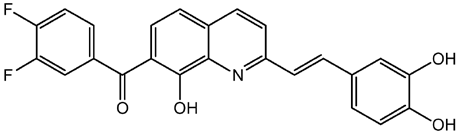	81.81± 8.80%	43.52± 0.73%
KHD291	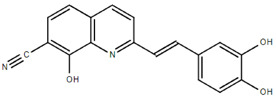	-68.13± 58.12%	71.17± 0.12%
KHD288	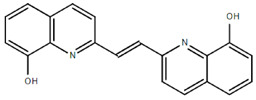	-8.41± 60.44%	62.68± 0.72%
MBN115	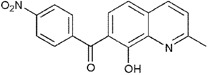	-400.96± 223.28%	77.08± 0.93
MD823	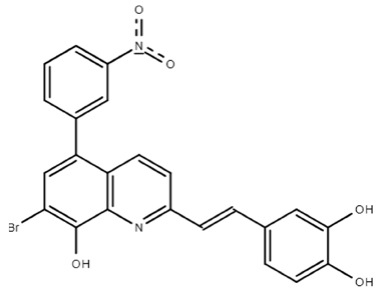	76.25± 3.16%?	58.86± 1.59%
FZ49	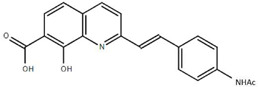	21.83± 28.39%	72.72± 1.22%
DD6	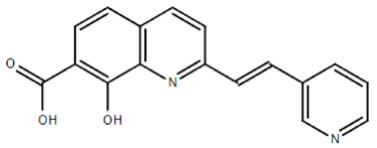	-8.49± 18.58%	96.73± 2.01%
DD7	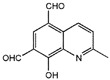	-31.56± 80.91%	90.32± 1.64%
DD8	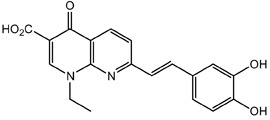	-74.03± 100.51%	91.54± 0.41%
FZ142	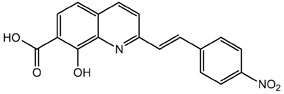	63.08± 2.64%	101.60± 1.39%
TOF411	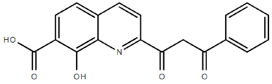	71.04± 0.67%	104.87± 0.60%
TOF401	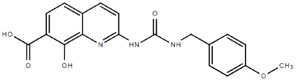	-44.62± 11.87%	106.21± 1.08%
MD20	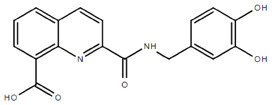	-124.41± 93.37%	108.35± 0.56%
MBN87	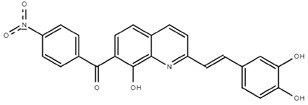	-17.91± 51.80%	70.50± 0.78%
MBN140	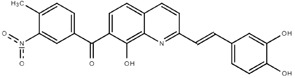	-68.13± 58.12%	67.58± 0.73%
FS48	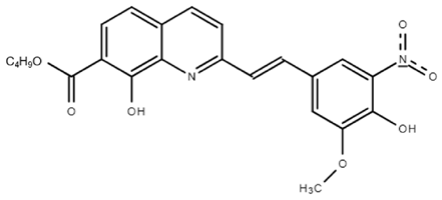	-8.41± 60.44%	81.47± 0.97%
AS65	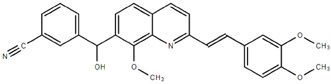	-10.17± 65.40%	77.51± 0.40%

The column on the left indicates the percent inhibition of trVLP replication using 10 µM, and the column on the right indicates the drug toxicity as assessed using MTT viability. **Inhibition:** HEK 293T cells were either transfected with replication machinery plasmids VP30, VP35, NP and Tim-1 (-L) or transfected with all replication machinery plasmids and Tim-1, allowing trVLP entry and replication (+L). Cells were treated with a 10µM final dose of each quinoline derivative, two hours pre-infection. Cells were infected with 100µL of viral stock diluted in 200µL of DMEM with 5% FBS. Cells were lysed, and luciferase activity was assessed 72 hours post-infection. Data represents three biological replicates. Data background corrected and displayed as a percentage of the positive control. Error shown is standard error of the mean. **Viability:** Drugs were added at a final dose of 10µM, 0.1% DMS. 26 hours later, media was replaced with 100µL of 0.5 mg/mL MTT in media without phenol red. After incubating for two hours at 37°C, 100 μL 10% SDS was added. MTT was fully dissolved at 37°C, then absorbance was read at 570nm. Wells containing no cells served as blanks. Cell viability was determined as percent absorbance of treatment to 0.1% DMSO control. Data shown represents four biological replicates. Data background corrected and displayed as a percentage of the no treatment control. Error shown is the standard error of the mean.

## Methods

### Drugs

Chloroquine diphosphate (Chl) was purchased from Abcam (catalog #ab142116) and amodiaquine dihydrochloride dihydrate (Amo) was purchased from Sigma-Aldrich (catalog #A2799) Artesunate (Art) was provided by the BioCIS research group at University Paris-Saclay, France. Novel quinoline derivatives: 2PentQ ^11, 12^, 2OHQ ^12^, 2NH2Q ^13^, 2COOHQ ^14^, 2CNQ ^15, 16^, 2QQ ^17^, 2Qi15 ^18^, 2Q16OH ^18^, 2QPOH ^19^, 2PQSel ^11^, DD1 ^20^, DD2 ^21^, DD3 (Desmaële unpublished; see *Extended data*) ^22^, DD4, (Desmaële unpublished; see *Extended data*) ^22^, DD5 (Desmaële unpublished; see *Extended data*) ^22^, MBN111 ^20^, MBN132 ^20^, MBN91 ^20^, KHD291 ^23^, KHD288 ^23^, MBN115 (Desmaële unpublished; see *Extended data*) ^22^, MD823 (Desmaële unpublished; see *Extended data*) ^22^, FZ49 ^24^, DD6 ^24^, DD7 (unpublished; see *Extended data*) ^22^, DD8 (Desmaële unpublished; see *Extended data*) ^22^, FZ142 ^24^, TOF411 ^25^, TOF401 ^25^, MD20 (Desmaële unpublished; see *Extended data*) ^22^, MBN87 ^20^, MBN140 ^20^, FS48 (Desmaële unpublished; see *Extended data*) ^22^, AS65 (Desmaële unpublished; see *Extended data*) ^22^, XF906 ^17^ and BS460 ^11^ were synthesized at University Paris-Saclay, France. Among them, 2PQsel and 2OHQ have exhibited *in vitro* and *in vivo* activities against the parasite *Leishmania* spp. ^26^.

### Cell culture

HEK-293T (American Type Culture Collection; ATCC, Rockville, USA) were grown in polystyrene coated, 75 cm ^2^ flasks (Sarstedt) in 15 mL of Dulbecco’s Modified Eagle Medium (DMEM) containing 10% FBS at 5% CO _2_ atmosphere and 37°C. Cells were harvested by washing using phosphate-buffered saline, followed by incubation with 2mL of trypsin/EDTA for 5 minutes in 5% CO _2_ atmosphere at 37°C.

### trVLP infection

To evaluate the efficacy of quinoline compounds of possible inhibition of Ebola virus, a replication competent mini-genome system developed by Hoenen *et al.* ^27^ was adopted. Transcription and replication competent viral-like particles (trVLP) are used to model the Ebola virus lifecycle. Within the trVLPs resides, a mini-genome containing structural viral proteins (VP24, VP40, and GP1,2) with the addition of a luciferase reporter gene. To facilitate viral replication, target cells (HEK-293T) are transfected with expression plasmids containing the remaining Ebola replication machinery proteins (VP30, VP35, NP, and L) 24 hours prior to infection. This model represents the most comprehensive lifecycle model available while still being able to be conducted in a biosafety level 2 facility ^27– 31^.

As described by Hoenen *et al.* ^27^, producer HEK-293T cells were seeded at 2.5 million cells/mL into 10cm Petri dishes in 10mL of DMEM containing 10% FBS at 5% CO _2_ atmosphere and 37°C. After 24 hours, cells were transfected with expression plasmids for the Ebola tetracistronic minigenome, virus replication machinery proteins, and bacteriophage T7 polymerase. The plasmids used have been previously described elsewhere ^29^ and were: tetracistronic minigenome; p4cis-vRNA-Rluc, T7 polymerase; pCAGGS-T7, viral protein 30; pCAGGS-VP30, viral protein 35;pCAGGS-VP35, viral NP; pCAGGS-NP, viral polymerase L; pCAGGS-L. All transfections were carried out using the calcium phosphate method (CalPhos Mammalian Transfection Kit, Clontech Laboratories; catalog #631312). Plasmids are described in Hoenen *et al.* ^27^. At 24 hours post-transfection, media was replaced with 15 mL of DMEM with 5% FBS. trVLPs containing supernatants were collected after 72 hours and stored at -80°C.

### Drug screen

To assess the efficacy of compounds for inhibition of viral replication *in vitro*, 40,000 293T cells, quantified using a hemocytometer, were seeded in 400µL of DMEM containing 10% FBS. After 24 hours, seeding cells were transfected with replication machinery expression plasmids as well as the cellular Ebola virus attachment factor Tim-1 (plasmid pCAGGS-Tim1) using the CalPhos Mammalian Transfection Kit (Clontech Laboratories; catalog #631312) ^27^. 24 hours following transfection, media was removed, followed by the addition of 300µL DMEM with 5% FBS. To this, 6µL of 100X drug stock prepared in 10% DMSO w/w was added. Two hours post-treatment, 100µL of viral stock diluted in 200µL of DMEM with 5% FBS, warmed to 37°C, was added to cells. The following day, media was replaced with 800µL of DMEM with 5% FBS. 72 hours post-infection, media was removed and cells were lysed in 200µL of 1x Renila Luciferase Assay Lysis Buffer (Renilla Luciferase Assay System, Promega; catalog #E2820). Luciferase activity in 20µL of lysate was measured using a Luminoskan Ascent microplate luminometer (Thermo Electron) after the addition of 100µL of Luciferase Assay Substrate diluted in Assay Buffer (Renilla Luciferase Assay System, Promega; catalog #E2820).

### MTT

Cells were seeded into 96-well plates at a density to mimic day two (post-transfection) stage (30% confluent). Drugs were added to the final dose, 0.1% DMSO, and 26 hours later, media was replaced with 100 μL 0.5 mg/mL 3-(4,5-dimethylthiazol-2-Yl)-2,5-diphenyltetrazolium bromide (MTT) in media without phenol red. After incubating for two hours at 37°C, 100μL 10% SDS was added. MTT was fully dissolved at 37°C, then absorbance was read at 570nm using a Biotech Epoch 2 spectrophotometer. Wells containing no cells served as blanks. Cell viability was determined as a percentage of absorbance of the background-corrected 0.1% DMSO treatment control.

### Statistics

Means were compared using the two-tailed, paired Student’s t-test (assuming equal variances) using Microsoft Excel, version 2016. A p-value ≤0.05 was considered significant. Error bars for Table 1 infection data are the range for n=2.

## Results

To assess the ability of quinolines to inhibit Ebola virus infection, we employed an established mini-genome model of Ebola replication, trVLP, allowing us to work under biosafety containment level 2 (CL 2) conditions ^27– 31^.

**Figure 1.**
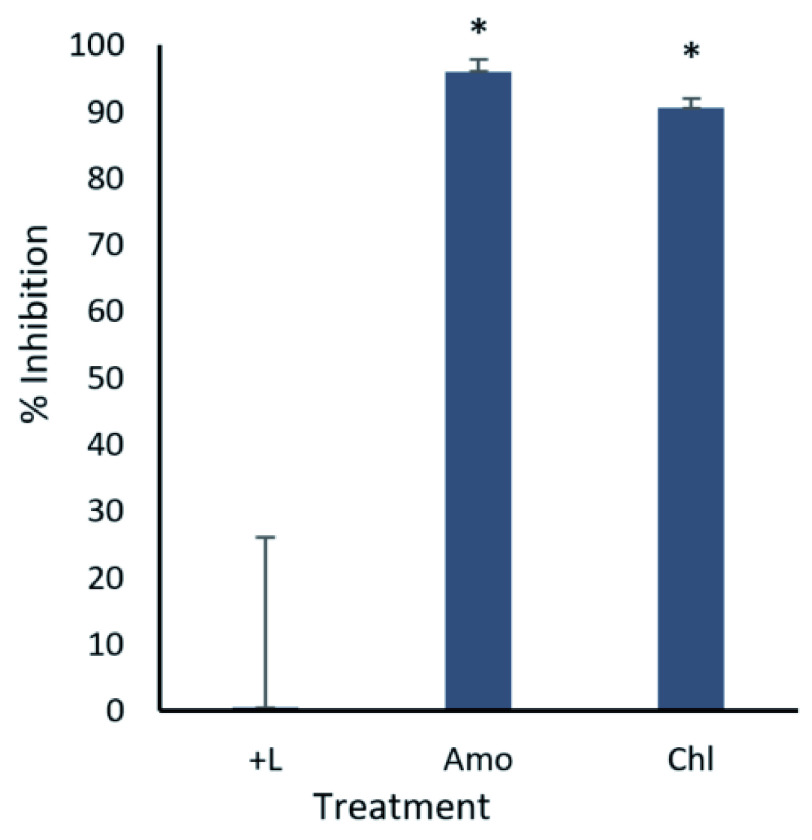
Amodiaquine and chloroquine inhibit trVLP replication. 293T cells were either transfected with replication machinery plasmids VP30, VP35, NP and Tim-1 (-L) or transfected with all replication machinery plasmids and Tim-1, allowing trVLP entry and replication (+L). Cells were treated with either 10µM amodiaquine or chloroquine two hours pre-infection or received no treatment. Cells were infected with 100µL of viral stock diluted in 200µL of DMEM with 5% FBS. Cells were lysed, and luciferase activity assessed 72 hours post-infection. Data shown represents four biological replicates. Data background corrected and displayed as a percentage of the positive control. Error bars shown are the standard error of the mean. Results from drug treatments were statistically compared with the (+) control group. * Denotes a p-value <0.05.

**Figure 2.**
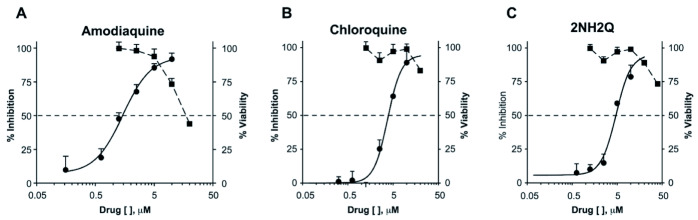
Chloroquine, amodiaquine and 2NH2Q inhibit trVLP in a dose dependent manor. **Circular markers, solid line:** 293T cells were either transfected with replication machinery plasmids VP30, VP35, NP and Tim-1 (-L) or transfected with all replication machinery plasmids and Tim-1, allowing trVLP entry and replication (+L). Cells were treated with either amodiaquine, chloroquine or 2NH2Q at concentrations of 10, 5, 2.5, 1.25, 0.625, or 0.31µM, two hours pre-infection. Cells were infected with 100µL of viral stock diluted in 200µL of DMEM with 5% FBS. Cells were lysed, and luciferase activity assessed 72 hours post-infection. Data shown represents four biological replicates. Data background corrected and displayed as a percentage of the positive control. Error bars shown are the standard error of the mean. **Square markers, dashed line:** 293T cells were seeded to mimic day two (post-transfection) stage (30% confluency). Drugs were added at a final dose of 0.47, 0.94, 1.88, 3.75, 7.5, 15, or 30 µM, 0.1% DMS. 26 hours later, media was replaced with 100µL of 0.5 mg/mL MTT in media without phenol red. After incubating for two hours at 37°C, 100 μL 10% SDS was added. MTT was fully dissolved at 37°C then absorbance was read at 570nm. Wells containing no cells served as blanks. Cell viability was determined as a percentage of the absorbance of treatment to 0.1% DMSO control. Data shown represents three biological replicates. Data background corrected and displayed as a percentage of the no treatment control. Error bars shown are the standard error of the mean.

In our first series of tests, the ability of amodiaquine and chloroquine to inhibit *in vitro* infection of Ebola virus replication was tested ( Figure 1). Following transfection with the required replication machinery and attachment receptor expression plasmids, HEK-293T cells were treated with the drugs at a concentration of 10 µM two hours pre-infection. Luciferase activity was measured 72 hours post-infection ^22^. Both amodiaquine and chloroquine demonstrated significant reductions in luciferase activity, indicating inhibited viral transcription and replication ^32^. Following these findings, dose-response experiments for amodiaquine and chloroquine were conducted. Inhibition activity was assessed at concentrations ranging from 10 µM to 0.31 µM ( Figure 2A and 2B). Amodiaquine exhibited a half maximal inhibitory concentration (IC _50_) of 1.45 µM, while chloroquine exhibited an IC _50_ of 3.95 µM. Cellular toxicity for both amodiaquine and chloroquine was measured using an MTT viability assay ( Figure 2A and 2B) to quantify drug toxicity along the concentration response ^22^.

To rapidly assess the inhibitory ability of the 36 novel quinoline derivatives ( Table 1), drugs were screened at a concentration of 10 µM as described above, with drug treatments occurring two hours pre-infection. From these tests, 2OHQ ^12^, 2NH2Q ^13^, 2QQ ^17^, 2Qi15 ^18^, MD823, TOF411, DD2, DD1, and MBN91 were identified as having inhibitory activity in our *in vitro* assay ( Table 1). However, due to drug toxicity or availability of synthesized drug, only 2NH2Q was pursued for further study. A dose-response for 2NH2Q was conducted, with inhibition being assessed between 10 µM and 0.31 µM ( Figure 2C). Results indicated the IC _50_ of 2NH2Q to be 4.66 µM, slightly higher than either amodiaquine or chloroquine.

## Discussion

As outbreaks of EVD continue to occur, treatment and preventative strategies are urgently needed. ****In August 2018, the Ministry of Health of the Democratic Republic of the Congo declared a new outbreak of Ebola virus disease in North Kivu Province. As of January 19, 2019, there have been 636 confirmed cases of EVD and 370 confirmed deaths ^2, 7^. Previously, it was suggested that anti-malarial drugs may be effective for the treatment of EVD ^8– 10^. In the report, we have provided supporting evidence that antiparasitic drugs can inhibit Ebola virus replication *in vitro,* which could be a breakthrough in fighting EVD if proven by additional studies or clinical trials.

Quinoline is a heterocyclic aromatic organic compound and is the base structure for many small molecule drugs. Quinoline-based drugs are widely used for the treatment of malarial infections. As their production is cheap, they are easy to synthesize with high yields, and are chemically stable while being readily accessible, quinolines offer many advantages for the treatment of infectious disease in tropical climates, such as the climates found in Africa ^8, 27^.

Our study aimed to test anti-malarial compounds against an Ebola replication model using the previously published trVLP *in vitro* assay, which has been shown to accurately reflect CL4 studies using intact infectious Ebola virus ^29^. We find amodiaquine to be effective at inhibiting Ebola viral replication. Amodiaquine is a useful anti-malarial agent in regions where Ebola virus outbreaks are possible, and this drug has been suggested to help with EVD outbreaks ^10^. We also show using our assay that other antiparasitic malarial drugs can inhibit Ebola virus replication with low cellular toxicity. These include chloroquine, 3/35 novel synthetic quinolines, one termed 2NH2Q, with high efficacy in inhibiting Ebola virus replication and low toxicity, and 4/35 showing moderate efficacy in inhibiting Ebola virus replication while being relatively non-toxic ( Table 1).

Quinolines, such as amodiaquine or chloroquine, have been used in regions at risk for EVD to prevent malaria and some have stated that use of these compounds is correlated with reduced death from EVD, suggesting that quinolines could be useful as both an anti-malarial and EVD treatment/prevention drug.

In summary, 38 compounds selected for their antiparasitic activity were evaluated against Ebola virus and their cytotoxicity was also determined. Some of them had antileishmanial activity, such as 2PQsel ^13^ and others exhibited antiplasmodial activity, such as chloroquine, amodiaquine and BS460 ^33^. The most interesting compounds emerging from viral inhibition and cytotoxicity assays were chloroquine and amodiaquine, with the advantage of chloroquine having no toxicity at 10 µM. From the quinoline series, the most promising compounds were 2Qi15 and 2NH2Q, both of them being 2-substituted quinolines. When studying structure-activity relationships, the presence of an amino group at the extremity of the propylene chain (compound 2NH2Q) allowed a better cell viability (81%) than a hydroxyl group (compound 2OHQ, 31%), despite their similar capacity for viral inhibition (89%). Replacement by a carboxylic group (compound 2COOHQ) strongly reduced the antiviral inhibition but maintained good cell viability. The other substitutions performed on the quinoline scaffold were not really satisfactory in regard to their biological activity.

 Our results support the hypothesis that antiparasitic quinolines active against malaria and leishmaniasis could be efficacious as a treatment for EVD. Further studies are necessary to confirm the utility of quinolines as anti- Ebola virus therapeutics, including consideration of these drugs when designing future Ebola drug trials in Africa.

## Data availability

### Underlying data

Harvard Dataverse: Inhibition of in vitro Ebola infection by anti-parasite quinoline derivatives. https://doi.org/10.7910/DVN/ONGV6I ^22^

This project contains the following underlying data:

- Underlying luciferase and MTT data for Figure 1 and Figure 2 and Table 1 (in XLSX format)
- Key to alternative compound names (in XLSX format)

### Extended data

Harvard Dataverse: Inhibition of in vitro Ebola infection by anti-parasite quinoline derivatives. https://doi.org/10.7910/DVN/ONGV6I ^22^

This project contains the following extended data:

- Supplemental data.DOCX (details of the synthesis of the unpublished compounds)

Data are available under the terms of the Creative Commons Zero "No rights reserved" data waiver (CC0 1.0 Public domain dedication).
