# Supplementary material for: Inhibition of in vitro Ebola infection by anti-parasitic quinoline derivatives
Source: F1000Res. 2020 Apr 17;9:268. [Version 1] doi: 10.12688/f1000research.22352.1 (PMC7268155; doi:10.12688/f1000research.22352.1)
Supplement: Peer review history

# Peer review: Reviewer response for version 1

**DOI:** 10.5256/f1000research.24661.r64097

**Published:** 2020-06-02

**Authors:** Jonas Šarlauskas

**Affiliations:** 1 Department of Xenobiotics Biochemistry, Institute of Biochemistry, Vilnius University, Vilnius, Lithuania

The authors of the current report investigated an *in vitro* activity of 36 anti-parasitic quinoline derivatives against Ebola infection. A study represents a novel, original and important information about significant antiviral activity of selected  quinoline compounds against this dangerous infection. To my mind, the paper is well written, general structure and length of the current manuscipt are fully acceptable and it is recommended to be indexed it in the present form.

Is the work clearly and accurately presented and does it cite the current literature?

Yes

If applicable, is the statistical analysis and its interpretation appropriate?

Yes

Are all the source data underlying the results available to ensure full reproducibility?

Yes

Is the study design appropriate and is the work technically sound?

Yes

Are the conclusions drawn adequately supported by the results?

Yes

Are sufficient details of methods and analysis provided to allow replication by others?

Yes

Reviewer Expertise:

Medicinal Chemistry, heterocyclic compounds

I confirm that I have read this submission and believe that I have an appropriate level of expertise to confirm that it is of an acceptable scientific standard.

# Peer review: Reviewer response for version 1

**DOI:** 10.5256/f1000research.24661.r62501

**Published:** 2020-05-27

**Authors:** Veljko Veljkovic

**Affiliations:** 1 Biomed Protection, Galveston, TX, USA

In this article, the authors investigated the repurposing of anti-malarial drugs for the treatment of Ebola virus disease. The presented study of anti-malarial quinolines showed that these compounds effectively inhibit Ebola virus replication in vitro. These results point out anti-parasitic quinoline compounds as a template for the development of novel safe and cheap drugs for the treatment of the Ebola disease. This clearly presented and experimentally well-supported manuscript is suitable for indexing in F1000Research.

Is the work clearly and accurately presented and does it cite the current literature?

Yes

If applicable, is the statistical analysis and its interpretation appropriate?

Not applicable

Are all the source data underlying the results available to ensure full reproducibility?

Yes

Is the study design appropriate and is the work technically sound?

Yes

Are the conclusions drawn adequately supported by the results?

Yes

Are sufficient details of methods and analysis provided to allow replication by others?

Yes

Reviewer Expertise:

Bioinformatics

I confirm that I have read this submission and believe that I have an appropriate level of expertise to confirm that it is of an acceptable scientific standard.
